# Mutation of the *Streptococcus gordonii* Thiol-Disulfide Oxidoreductase SdbA Leads to Enhanced Biofilm Formation Mediated by the CiaRH Two-Component Signaling System

**DOI:** 10.1371/journal.pone.0166656

**Published:** 2016-11-15

**Authors:** Lauren Davey, Scott A. Halperin, Song F. Lee

**Affiliations:** 1 Department of Microbiology and Immunology, Dalhousie University, Halifax, NS, B3H 1X5, Canada; 2 Canadian Center for Vaccinology, Dalhousie University and the IWK Health Centre, Halifax, NS, B3K 6R8, Canada; 3 Department of Pediatrics, Faculty of Medicine, Dalhousie University and the IWK Health Centre, Halifax, NS, B3K 6R8, Canada; 4 Department of Applied Oral Sciences, Faculty of Dentistry, Dalhousie University, Halifax, NS, B3H 4R2, Canada; LSU Health Sciences Center School of Dentistry, UNITED STATES

## Abstract

*Streptococcus gordonii* is a commensal inhabitant of human oral biofilms. Previously, we identified an enzyme called SdbA that played an important role in biofilm formation by *S*. *gordonii*. SdbA is thiol-disulfide oxidoreductase that catalyzes disulfide bonds in secreted proteins. Surprisingly, inactivation of SdbA results in enhanced biofilm formation. In this study we investigated the basis for biofilm formation by the *ΔsdbA* mutant. The results revealed that biofilm formation was mediated by the interaction between the CiaRH and ComDE two-component signalling systems. Although it did not affect biofilm formation by the *S*. *gordonii* parent strain, CiaRH was upregulated in the *ΔsdbA* mutant and it was essential for the enhanced biofilm phenotype. The biofilm phenotype was reversed by inactivation of CiaRH or by the addition of competence stimulating peptide, the production of which is blocked by CiaRH activity. Competition assays showed that the enhanced biofilm phenotype also corresponded to increased oral colonization in mice. Thus, the interaction between SdbA, CiaRH and ComDE affects biofilm formation both *in vitro* and *in vivo*.

## Introduction

*Streptococcus gordonii* is a commensal inhabitant of human oral cavity. It is non-cariogenic, and its presence is associated with oral health [1,2]. Colonization with *S*. *gordonii* is beneficial because it can neutralize the surrounding pH to mitigate damage from cariogenic species, in addition to directly inhibiting the growth of some pathogens by secreting substances such as bacteriocins and hydrogen peroxide [3–7]. More passively, it also occupies space in the oral biofilm that would otherwise be available to cariogenic species, such *Streptococcus mutans*.

As a pioneer colonizer, *S*. *gordonii* colonizes early in life and is able to bind directly to salivary proteins on the tooth surface, forming the base of oral biofilms [8,9]. The ability to adhere and form biofilms in the host is crucial for persistence in the oral cavity; otherwise *S*. *gordonii* would be washed away. In addition, biofilms increase fitness by facilitating natural genetic transformation and by providing a protective niche in the continually fluctuating environment of the oral cavity [8,10,11]. Biofilm formation by *S*. *gordonii* is a complex process involving adhesins, signalling systems, ABC-transporters, and glycosyltransfrases among other factors [12–16]. These factors cooperate to maintain biofilms in the competitive and stressful environment of the oral cavity.

Recently we found that an enzyme required for disulfide bond formation, SdbA, played a role in biofilm formation [17]. SdbA is a thiol-disulfide oxidoreductase that catalyzes disulfide bond formation in extracytoplasmic proteins [17,18]. These bonds are important for the folding and stability of certain proteins, and *ΔsdbA* mutants are unable to form disulfide bonds. This generates a stress signal that triggers activation of the two-component signalling system CiaRH, presumably in response to an accumulation of misfolded proteins [19]. CiaH is a histidine kinase located at the membrane that activates the response regulator CiaR, which then drives the expression of multiple proteins including DegP (HtrA), a quality control protease that degrades aberrant proteins at the cell surface [19,20].

Inactivation of *sdbA* generates a pleiotropic phenotype [20]. The mutants are deficient in genetic competence, bacteriocin production, and extracellular DNA (eDNA) production, and autolysis, yet, somewhat surprisingly inactivation of *sdbA* enhances biofilm formation. Some of these phenotypes are a direct result of inactivation of *sdbA*. For example, the major autolysin AtlS is a natural substrate of SdbA, and is therefore inactive in the *ΔsdbA* mutant. Other phenotypes, such as the loss of bacteriocin production, are a stress response mediated by CiaRH. Bacteriocin production in *S*. *gordonii* is regulated by the ComDE quorum-sensing system. The histidine kinase ComD is activated when it senses an accumulation of secreted competence-stimulating peptide (CSP), and upon activation, it phosphorylates the response regulator ComE. This ultimately leads to expression of the bacteriocin genes, as well as genetic competence. However, activation of CiaRH in the *ΔsdbA* mutant eliminates CSP production, effectively shutting down the ComDE pathway and bacteriocin production [19]. Thus the pleiotropic phenotype of the *ΔsdbA* mutant involves multiple mechanisms, some of which are not directly related to disulfide bond formation.

The basis for enhanced biofilm formation by the *ΔsdbA* mutant is unknown. In this study, we sought to investigate how inactivation of *sdbA* leads to the hyperbiofilm phenotype, and to determine the effect of SdbA on oral colonization in mice. Our results reveal that biofilm formation by the *ΔsdbA* mutant is mediated by the CiaRH two-component signalling system, and the ability of CiaRH to repress production of competence stimulating peptide (CSP).

## Results

### CiaRH expression in biofilms

Previously, we found that expression of the two-component signalling system CiaRH is upregulated in the *ΔsdbA* mutant [19]. Although the role of CiaRH in biofilm formation by *S*. *gordonii* has not been investigated, CiaRH is required for biofilm formation and colonization in other streptococci, including *S*. *pneumoniae* [21–25], *S*. *mutans* [26], and group B *Streptococcus* [27]. This suggested that upregulation of CiaRH in the *ΔsdbA* mutant might contribute to its enhanced biofilm phenotype.

Our previous investigation of *ciaRH* expression in the *ΔsdbA* mutant examined cultures grown in BHI to the early exponential growth phase, which coincides with bacteriocin production and natural genetic competence. To determine if *ciaRH* expression is also upregulated when the *ΔsdbA* mutant is grown in biofilms, we tested expression using cells prepared from either the biofilm inoculum or 24 h biofilms.

**Fig 1:**
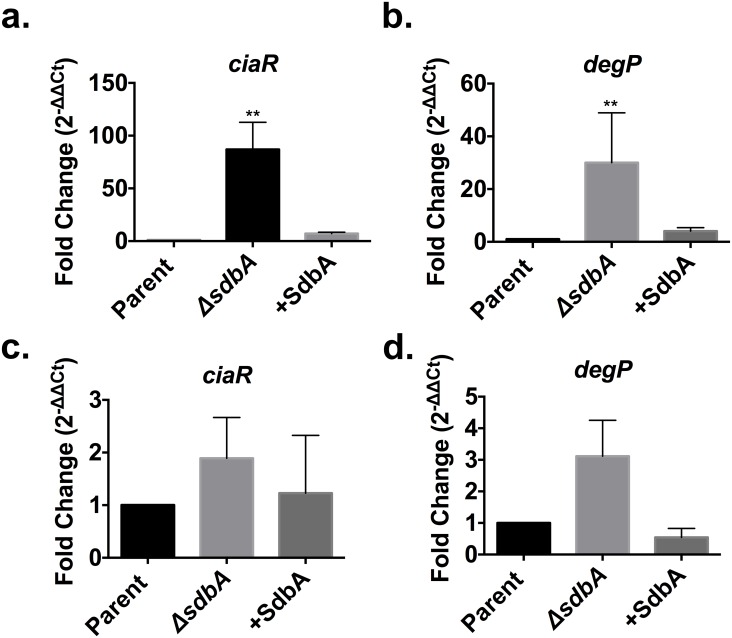
CiaRH expression in the *S*. *gordonii* biofilms. Expression of the *cia*-induced genes *ciaR* and *degP* in the biofilm inoculum and in 24 h biofilms. (a) Expression of *ciaR* in the parent, *ΔsdbA* mutant, and *sdbA*-complemented mutant (+SdbA) in the biofilm inoculum. RNA was isolated from cells grown to early stationary phase in HTVG medium. (b) Expression of *degP* in the biofilm inoculum. Biofilms were grown in a defined biofilm medium (BM), and RNA was isolated from biofilm cells grown in BM medium for 24 h. (c) Expression of *ciaR* and (d) *degP* in 24 h biofilms. Results are means ± SD of three experiments. Data were analyzed by one-way ANOVA and asterisks indicate a significant difference from the parent (**P < 0.01).

Biofilms were grown following the protocol described by Loo *et al*., (42), which was designed to optimize *S*. *gordonii* biofilm formation *in vitro*. This approach uses cells grown in a rich medium as the inoculum, and a defined biofilm medium (BM) for subsequent biofilm growth. To investigate *ciaRH* expression and biofilm formation by the *ΔsdbA* mutant, biofilm inoculum was prepared from cells grown to the early stationary phase in HTVG medium. Analysis of the inoculum revealed that *ciaR* expression was upregulated by an average of >80-fold in the *ΔsdbA* mutant compared to the parent (Fig 1a). Expression of *degP*, a serine protease regulated by CiaRH, was also upregulated by an average of 30-fold, supporting the notion that the CiaRH system is activated in the *ΔsdbA* mutant (Fig 1b). After the cells had been grown in biofilms for 24 h, *ciaR* and *degP* expression in the *ΔsdbA* mutant biofilms had decreased to a level that was not significantly different from the parent, although expression remained 2 to 3-fold higher in the *ΔsdbA* mutant compared to the parent (Fig 1c and 1d). This suggested that CiaRH signalling might influence biofilm formation by the *ΔsdbA* mutant, particularly during the initial phases of biofilm development.

### Biofilm formation by the *ΔsdbA* mutant is mediated by CiaRH

**Fig 2:**
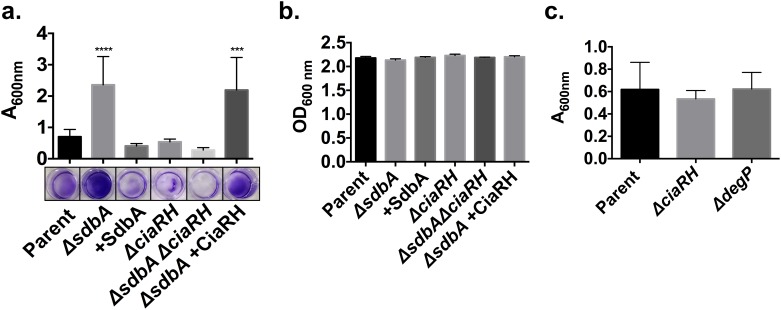
CiaRH is required for biofilm formation by the *ΔsdbA* mutant. (a) Crystal violet staining of 24 h biofilms grown in 24-well plates. Biofilms were grown with the parent, *ΔsdbA*, *sdbA*-complemented mutant (+SdbA), *ΔciaRH*, *ΔsdbAΔciaRH* double mutant, and *ΔsdbA ciaRH*-complemented mutant (*ΔsdbA* + CiaRH). Results are means ± SD of at least three experiments. The lower panel shows representative wells after staining. (b) In parallel to the biofilm formation assay, three additional wells for each strain were tested for total growth. The optical density was measured for the combined biofilm and planktonic cells for each mutant. (c) Biofilm formation of single deletion mutants for *ciaRH* and *degP* in the parent strain. Biofilms were grown for 24 h in 24-well plates prior to staining. Data were analyzed by one-way ANOVA and asterisks indicate a significant difference from the parent (***P ≤ 0.001, ****P ≤ 0.0001).

To determine if upregulation of *ciaRH* in the *ΔsdbA* mutant contributed to biofilm formation, we tested biofilm formation in CiaRH-deficient mutants. Analysis of 24 h biofilms revealed that CiaRH was critical for biofilm formation by the *ΔsdbA* mutant. Mutation of *ciaRH* abolished the enhanced biofilm phenotype in the *ΔsdbA* mutant, while complementation with a functional ciaRH system reversed the phenotype (Fig 2a) (*P* <0.0001). Although the *ΔsdbAΔciaRH* mutant formed very little biofilm, it did not have an obvious growth defect, and the total growth (planktonic plus biofilm cells) was similar across all of the mutants (Fig 2b).

Surprisingly, however, inactivation of *ciaRH* and *degP* in the parent did not affect biofilm formation (Fig 2a and 2c). This suggested that the basal level of CiaRH activity in the parent is not a major contributor to biofilm formation, whereas upregulation of *ciaRH* is crucial for biofilm formation by the *ΔsdbA* mutant.

### Biofilm formation by *S*. *gordonii ΔsdbA* and *ΔsspAB* mutants involve different mechanisms

Reports of mutations that enhance biofilm formation in *S*. *gordonii* are rare. One exception are mutants that lack the antigen I/II proteins SspA and SspB. Similar to the *ΔsdbA* mutant, the *ΔsspAΔsspB* mutant displays increased biofilm formation and initial attachment [14]. The enhanced biofilm formation by the *ΔsspAΔsspB* mutant has been attributed in part to upregulation of the surface lipoprotein ScaA, a substrate binding protein, which also enhances biofilm formation by *S*. *gordonii* in response to nicotine [14,28]. Initially we hypothesized that a similar scenario could be playing out in the *ΔsdbA* mutant, however, we found that *scaA* expression was unchanged in the *ΔsdbA* mutant (S1a Fig). In addition, we found that inactivation of the *ciaRH* system did not affect biofilm formation by the *ΔsspAΔsspB* mutant (S1b Fig). Thus, despite their similar phenotypes, biofilm formation by the *ΔsdbA* mutant involves a different mechanism.

**Fig 3:**
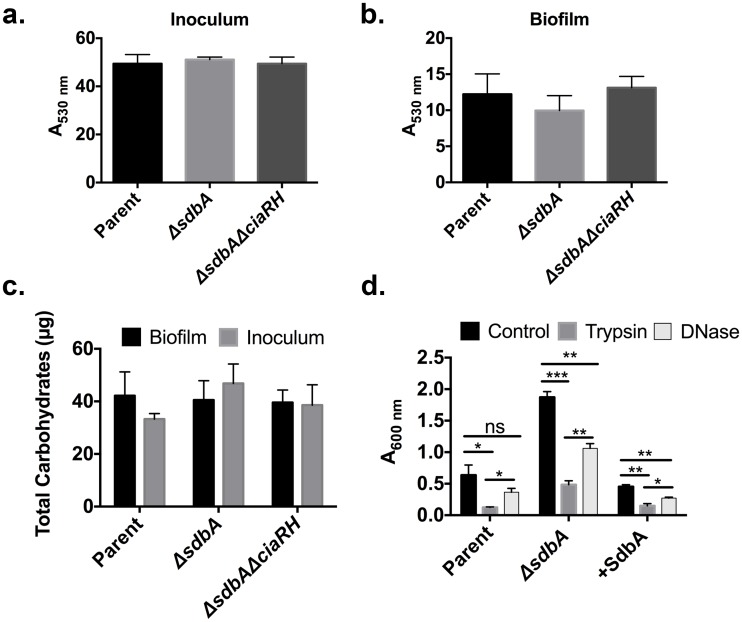
Characterization of *ΔsdbA* biofilms. (a) Alcian blue binding assay for surface charge in cells grown to early stationary phase in HTVG medium (Inoculum), and (b) in cells grown in 24 h biofilms (Biofilm). Bars represent the percentage of unbound dye. (c) Total carbohydrate production. (d) Sensitivity to DNase I and trypsin. Biofilms were grown for 24 h prior to the addition of either DNase I or trypsin, and incubated for an additional 1 h before staining with crystal violet. Data were analyzed by one-way ANOVA and asterisks indicate a significant difference from the control biofilm for each strain (***P ≤ 0.001**P < 0.01, *P < 0.05).

Multiple factors contribute to biofilm formation, including extracellular polysaccharides, eDNA, and proteins [12]. To get clues to the nature of the *ΔsdbA* biofilms, we tested total carbohydrate production, surface charge, and sensitivity to DNase I and trypsin. Surface charge and carbohydrate production of cells from the biofilm inoculum and 24 h biofilms was similar in all strains (Fig 3). DNase I treatment produced a modest decrease in the amount of biofilm, suggesting a minor role for eDNA in biofilm stability (Fig 3d). In contrast, trypsin significantly reduced biofilm formation in both the parent and the *ΔsdbA* mutant. Thus, proteins appear to be the most important factor contributing to biofilm formation and stability in *S*. *gordonii* (Fig 3d). The nature of the biofilm matrix appears to be similar between the *ΔsdbA* mutant and the parent, except that the total amount of surface attachment and biofilm formation is greater in the mutant. Although speculative, it is possible that certain proteins contributing to biofilm formation might be upregulated in response to the *sdbA* mutation.

### CiaRH mediated repression of the Com system contributes to biofilm formation by the *ΔsdbA* mutant

Previously we have shown that CiaRH represses the ComDE quorum-sensing system in the *ΔsdbA* mutant. ComD is a sensor histidine kinase that, along with its response regulator ComE, regulates expression over 150 genes in *S*. *gordonii*, including the genes for genetic competence and bacteriocin production [29]. ComDE is activated by secreted CSP, which is encoded by *comC*. Considering the relationship between SdbA, CiaRH, and ComDE, we hypothesized that these systems might contribute to biofilm production in the *ΔsdbA* mutant.

CiaRH represses the ComDE quorum sensing system in the *ΔsdbA* mutant by inhibiting production of the CSP autoinducer [19]. Accordingly, de-repression of ComDE can be achieved in two ways: (1) inactivate the CiaRH system or (2) expose cells to exogenous CSP [19]. Given that inactivation of CiaRH reduced biofilm formation in the *ΔsdbA* mutant, we asked whether exogenous CSP would produce a similar effect. In a previous study, we demonstrated that exogenous CSP induces *comCDE* expression in the *ΔsdbA* mutant to a level comparable to the parent [19].

**Fig 4:**
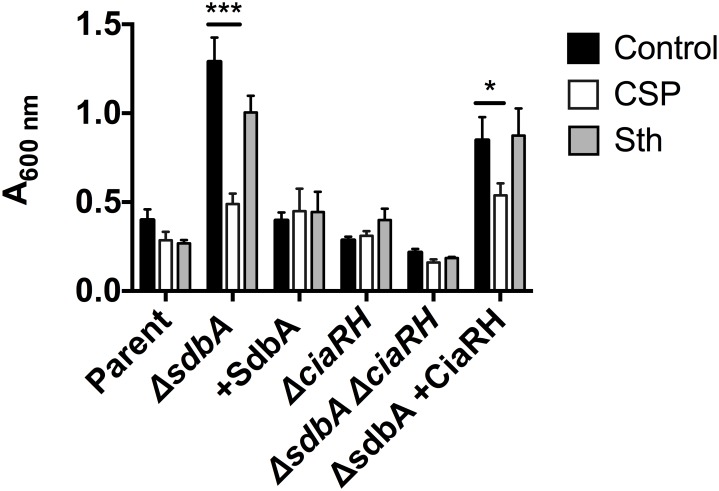
CSP diminishes biofilm formation by the *ΔsdbA* mutant. Crystal violet staining of 24 h biofilms grown in the presence of either CSP, Sth_1_ bacteriocin (Sth), or without added peptide (Control). Biofilms were grown with the parent, *ΔsdbA*, *sdbA*-complemented mutant (+SdbA), *ΔciaRH*, *ΔsdbAΔciaRH* double mutant, and *ΔsdbA ciaRH*-complemented mutant (*ΔsdbA* +CiaRH). Results are means ± SD of three experiments. Asterisks indicate a significant difference from the control biofilm for each strain, as determined by one-way ANOVA (****P < 0.0001, *P < 0.05).

To test the effect of the CSP on biofilm formation, biofilms were grown for 24 h in the presence of 0.5 μg/ml CSP and quantified by crystal violet staining. Exogenous CSP did not affect biofilm formation by the parent, which is consistent with previous findings [30]. However, when the *ΔsdbA* mutant was grown with exogenous CSP biofilm formation was reduced to a level that was not significantly different from the parent (Fig 4) (*P* <0.001). This effect was specific to the *ΔsdbA* mutant, and required a functional and activated CiaRH system. Consequently, CSP did not affect biofilm formation by the *sdbA*-complemented mutant or the *ΔsdbAΔciaRH* mutant. Similarly, exogenous CSP did not affect biofilm formation by the *ΔciaRH* single mutant. Complementation of *ciaRH* into the *ΔsdbAΔciaRH* background restored sensitivity to CSP.

CSP is a small charged peptide with 19 amino acids and a pI of 10.28. To determine if the biofilm reducing effect was specific to CSP, we tested the effect of another peptide, the *S*. *gordonii* bacteriocin Sth_1_ (17 amino acids, pI 12.01). Growth with Sth_1_ did not produce a significant decrease in biofilm formation by any of the strains (Fig 4). This indicates that the effect is specific to CSP, and suggests that CiaRH mediated repression of CSP production is required for biofilm formation by the *ΔsdbA* mutant.

### Inactivation of SdbA enhances oral colonization in mice

Although *in vitro* biofilm formation can vary depending on growth conditions and medium, it has been demonstrated to be correlated with *in vivo* colonization in mice in *S*. *pneumoniae*, including the role of CiaRH [21]. As an inhabitant of oral biofilms, biofilm formation is integral to *S*. *gordonii* colonization. The enhanced biofilm phenotype of the *ΔsdbA* mutant suggested that it might also affect colonization.

**Fig 5:**
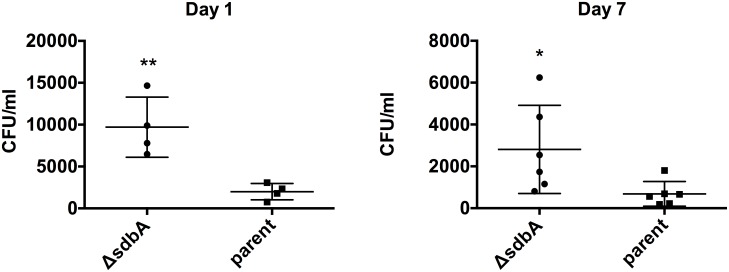
The *ΔsdbA* mutant outcompetes the parent an oral colonization model. Mice were inoculated with a 1:1 mixture consisting of ~10^9^ CFU of the parent and *ΔsdbA* mutant. Bacteria were recovered by swabbing oral surfaces after 1 and 7 days and enumerated using selective agar. Points represent the CFU/ml for the parent and the *ΔsdbA* mutant recovered from each mouse. Asterisks indicate a significant difference from zero using a one-sample T-test (**P < 0.01).

To determine how SdbA affects colonization, we ran a competitive assay between the parent and *ΔsdbA* mutant in a mouse model of oral colonization. We did not pursue colonization assays using the *ΔsdbAΔciaRH* mutant given its inability to form biofilm *in vitro*. Mice were inoculated with equal amounts of the parent and the *ΔsdbA* mutant. Following colonization, bacteria were recovered from the oral cavity and enumerated by growing with antibiotics that allowed differentiation of the two strains. Samples were plated on medium with tetracycline and spectinomycin to prevent growth of other oral bacteria, and with or without erythromycin to select for the *sdbA* mutant. The number of CFU for the parent was determined by subtracting the erythromycin resistant colonies from the total. Consistent with the ability of the *ΔsdbA* mutant to form more biofilm *in vitro*, the *ΔsdbA* mutant exhibited enhanced colonization after 24 h, with an average of 9.3 x 10^3^ CFU/ml compared to 2 x 10^3^ CFU/ml for the parent (*P* = 0.006) (Fig 5). By day 7, the difference was less pronounced and *S*. *gordonii* numbers were lower overall, however, the *ΔsdbA* mutant continued to outcompete the parent with an average of 2.8 x 10^3^ CFU/ml compared to 6.8 x 10^2^ CFU/ml (*P* = 0.039). Thus, the *ΔsdbA* mutant exhibits enhanced biofilm formation both *in vitro* and *in vivo*.

## Discussion

In this study we investigated the basis for enhanced biofilm formation by the *S*. *gordonii ΔsdbA* mutant. SdbA catalyzes disulfide bond formation in secreted proteins, but it was not clear how this impacted biofilm formation. The role of SdbA in biofilm formation appears to be primarily indirect, and mediated by the interaction between the CiaRH two-component signalling system and the Com quorum-sensing system.

Since disulfide bonds are important for the folding and stability of extracytoplasmic proteins, inactivation of a disulfide catalyst can affect multiple proteins on the cell surface. This has the potential to alter surface adhesion and biofilm initiation, in addition to creating a stress response. For example, inactivation of the disulfide bond forming enzymes DsbA or DsbB eliminates biofilm formation in pathogenic *E*. *coli* and *Pseudomonas aeruginosa* [31–33]. In some instances, however, biofilm formation can be enhanced, such as in *Pseudomonas putida* where DsbA mutants form more biofilm due to increased EPS production [34], or in *Salmonella enterica* DsbA mutants, which have enhanced biofilm formation as a result of increased production of the fimbriae protein CsgA [35]. Although the mechanisms are not fully understood, the increased protein production and biofilm formation appear to be a stress response involving stress-induced signalling systems [35].

We found that a similar situation occurs in the *S*. *gordonii ΔsdbA* mutant. Inactivation of SdbA generated a signal that triggered the stress-related two-component regulatory system, CiaRH, which in turn stimulated biofilm formation. The CiaRH system is found throughout the genus *Streptococcus* and is best characterized in *S*. *pneumoniae* where it contributes to cell wall homeostasis, antibiotic resistance, and repression of genetic competence [20,36–38]. CiaRH is also important for biofilm formation in *S*. *pneumoniae* and *S*. *mutans*, and similar to the findings presented here, upregulation of CiaRH in *S*. *pneumoniae* variants results in enhanced biofilm formation [21,24,26]. The system is not as well characterized in *S*. *gordonii*, although it generally appears to have similar properties, and has been shown to contribute to acid stress resistance and competence repression [19,39]. In addition, *ciaR* (SGO_1072) has been found to be upregulated when *S*. *gordonii* is grown in mixed biofilms with *Fusobacterium nucleatum* [40]. Our results suggest that CiaRH was crucial for biofilm formation by the *ΔsdbA* mutant, but was dispensable in the parent. A possible explanation for this observation is that CiaRH has a greater influence on biofilm formation under adverse conditions, such as in the fluctuating environment of the oral cavity, and additional investigation is required to better understand the role of CiaRH in the parent.

In a previous study, we found that *ciaRH* expression was upregulated approximately 4-fold in the *ΔsdbA* mutant when grown under conditions that induce bacteriocin production, which occurs during a brief window in the early exponential growth phase [19]. Here, we found that the *ΔsdbA* mutant upregulates *ciaRH* expression by approximately 80-fold compared to the parent when grown to early stationary phase in HTVG medium. This increase appeared to be critical for biofilm formation, although *ciaRH* expression levelled off once the cells were in biofilms, suggesting that expression is influenced by the growth phase and the medium used in the experiment. *ciaRH* expression in *S*. *pneumoniae* is influenced by growth conditions, and has been demonstrated to peak during the early stationary growth phase [36]. The results presented here indicate that *ciaRH* expression in *S*. *gordonii* might follow a similar pattern.

Our analysis of the *ΔsdbA* mutant biofilms suggested that the surface charge and EPS production is similar to the parent. Although CiaRH regulates *dlt* expression in *S*. *pneumoniae*, which affects the surface charge through D-alanylation of lipoteichoic acid, *ciaRH* does not affect *dlt* expression in *S*. *gordonii* [40]. Our results support this conclusion, since even a dramatic increase in *ciaRH* expression did not have a significant impact on surface charge. Similarly, eDNA was not a major contributor to the *ΔsdbA* mutant biofilm, which is consistent with our earlier observation that the *ΔsdbA* mutant produces less eDNA than the parent [17]. Rather, the enhanced biofilm phenotype of the *ΔsdbA* mutant was primarily dependent on extracytoplasmic protein (Fig 3c).

Biofilm formation by *S*. *gordonii* involves multiple factors, including the glucosyltransferase GtfG [41], two-component signalling systems [16,42], ABC-transporters [16], and multiple surface adhesins that facilitate binding to host surfaces and salivary proteins [14]. In some instances, deletion of certain surface proteins can lead to enhanced biofilm and colonization, such as has been observed in mutants lacking SspAB [14] and AbpA [41,43]. Although the surface lipoprotein ScaA has been linked to enhanced biofilm formation, *scaA* expression was not upregulated in the *ΔsdbA* mutant. Similarly, we did not detect differences between the protein profiles of the *ΔsdbA* mutant and the biofilm defective *ΔsdbAΔciaRH* mutant that would suggest changes in the abundance of a large surface adhesin (data not shown).

Instead, the mechanism of CiaRH mediated biofilm formation by the *ΔsdbA* mutant involves the Com quorum-sensing system. CiaRH negatively regulates the Com system in the *ΔsdbA* mutant by inhibiting CSP production, however the system remains functional and can respond to exogenous CSP [19]. The results presented here show that exogenous CSP inhibits biofilm formation by the *ΔsdbA* mutant. Previously, we demonstrated that exogenous CSP does not decrease CiaRH expression in the *ΔsdbA* mutant, and therefore biofilm inhibition by CSP is not related to altered CiaRH levels [19]. Although we can only speculate as to how CSP affects biofilm formation, there are at least two plausible mechanisms. First, it could be a direct effect of the CSP protein. Charged peptides can bind to the cell surface or to matrix components resulting in altered adhesion [44], and pneumococcal CSP was recently shown to be retained at the cell surface by ComD [45]. A second possible mechanism is that CSP activates Com signalling, which in turn affects expression of other factors that mediate biofilm formation. Considering that CSP influences expression of >100 genes, we think that this is the most probable scenario [29].

In addition to regulating genetic competence, ComDE is a global regulator that plays an important role in stress responses. An analysis of the transcriptional response to CSP in *S*. *gordonii* revealed 162 upregulated and 89 downregulated genes, many of which function in processes outside of the competence pathway [29]. The ComDE quorum-sensing system has been linked to biofilm formation and colonization in multiple species of streptococci, although its precise role remains unclear. For example, mutation of *comD* inhibits biofilm formation and colonization by *S*. *gordonii*, *S*. *mutans*, and *S*. *pneumoniae* [22,42,46], whereas mutation of *comE* alone actually enhanced colonization by *S*. *pneumoniae* [22]. It was hypothesized that the unphosphorylated form of *comE* inhibited attachment, and therefore attachment was increased in the *ΔcomE* mutant [22]. There is also evidence to suggest that CSP affects biofilm formation. For example, CSP enhances biofilm formation by *Streptococcus intermedius* [47]. In both *S*. *gordonii* and *S*. *mutans*, mutation of the *ΔcomCDE* operon, or *ΔcomC* (which encodes CSP), altered the biofilm density and architecture [30,46]. Although the *S*. *gordonii ΔcomC* mutation did not effect the total biomass of *S*. *gordonii* monoculture biofilms, the mutant formed more biofilm when grown in mixed cultures with the fungus *Candida albicans* [30]. Thus ComDE can clearly influence biofilm formation, but the relationship is complex, and not yet fully understood.

The enhanced biofilm phenotype of the *ΔsdbA* mutant appears to require a coordinated increase in CiaRH activity and repression of *comDE*. Although the mechanisms remain unclear, the interconnectedness of the CiaRH and ComDE systems, particularly in relation to stress responses, has been observed previously in multiple streptococci including *S*. *gordonii* [22,38,39,48,49]. This is exemplified by *S*. *pneumoniae*, where activation of genetic competence in CiaRH mutants causes the cells to lyse [38]. Although the degree of activation varies with growth conditions, we found that the CiaRH system is consistently upregulated in *S*. *gordonii ΔsdbA* mutants. CiaRH is associated with cell wall integrity, and provides protection from conditions that perturb the cell wall such as acid stress, β-lactam antibiotics, mutations in penicillin-binding proteins [24,26,39,49]. In the absence of SdbA, substrates requiring disulfide bonds will remain in a reduced state and are vulnerable to oxidation and misfolding. Since an accumulation of misfolded protein at the cell surface is a potentially lethal situation, upregulation of ciaRH, and the CiaR-regulated protease DegP, likely serves to mitigate stress from misfolded proteins in the *ΔsdbA* mutant.

In addition to a general stress response to misfolded proteins, CiaRH might also respond more directly to inactivation of the major autolysin AtlS, which is a natural SdbA substrate [17]. The *ΔsdbA* mutant produces AtlS protein, but it lacks autolytic activity and shows altered processing compared to the parent [17]. At this point, we can only speculate as to how mutation of SdbA triggers the activation of the CiaRH system, however, the loss of AtlS activity would be a plausible mechanism given its role in cell wall turnover. Notably, AtlS contributes to cell surface biogenesis and biofilm formation, and its expression is induced by exogenous CSP [50]. Thus, AtlS is a possible link between cell wall integrity, biofilm formation, and CSP. However, additional investigation will be required to understand how the inactive form of AtlS produced by the *ΔsdbA* mutant affects the cell, and whether it contributes to CiaRH mediated biofilm formation

Consistent with the enhanced biofilm formation observed *in vitro*, the *ΔsdbA* mutant also outcompeted the parent in a mouse oral colonization assay. We do not know if cross-contamination of CSP produced by the parent affected colonization by the *ΔsdbA* mutant in the competition assay, but it is plausible that most of the CSP would be washed away by saliva. Regardless, the mutant outcompeted the parent after 1 day, and continued to outnumber the parent after 7 days.

In summary, we investigated how mutation of a disulfide bond forming catalyst, SdbA, leads to enhanced biofilm formation by *S*. *gordonii*. The mechanism appears to be a general stress response mediated by the CiaRH two-component signalling system. Upregulation of CiaRH represses expression of the ComDE quorum-sensing system, which in turn leads to enhanced biofilm formation. Conversely, exposure to CSP reverses the phenotype. The interplay between the CiaRH and Com systems enhances both *in vitro* biofilm formation and *in vivo* colonization by the *ΔsdbA* mutant. *S*. *gordonii* is associated with oral health, and it has potential biotechnological value as a live vaccine vector, or possibly even as a probiotic [51]. The enhanced biofilm formation and colonization of the *ΔsdbA* mutant suggests that it could be particularly useful for such applications.

The interaction between multiple regulatory systems highlights the complexity of biofilm formation by *S*. *gordonii*. Understanding these pathways can provide a basis for investigating more complex biofilm communities and understanding how this commensal bacterium contributes to oral health.

## Methods and Materials

### Bacterial strains and growth conditions

Experiments were carried out using *S*. *gordonii* SecCR1 as the parent strain. *S*. *gordonii* SecCR1 is a derivative of *S*. *gordonii* Challis DL-1 that secretes a single-chain variable fragment antibody against complement receptor 1 (CR1), which is a protein that requires disulfide bonds for stability [52]. This strain has been used in our previous studies of disulfide bond formation in *S*. *gordonii*, and the *S*. *gordonii* SecCR1 *ΔsdbA* mutant has the same phenotype as the *S*. *gordonii* Challis DL-1 *ΔsdbA* mutant [19]. Additional strains and mutants are described in Table 1. *S*. *gordonii* was grown in either HTVG medium (0.5% (wt/vol) glucose, 3.5% (wt/vol) tryptone, 100 mM HEPES, 0.29 μM *p*-aminobenzoic acid, 0.59 μM thiamine-HCl, 8.2 μM nicotinamide, and 0.53 μM riboflavin, pH 7.6) [53], or in a semi-defined Biofilm Medium (BM; 58 mM K_2_HPO_4_, 15 mM KH_2_PO_4_, 10 mM (NH_4_)_2_SO_4_, 35 mM NaCl, 0.8% (wt/vol) glucose, 0.2% (wt/vol) Casamino Acids, 25 μM MgCl_2_7H_2_O, 2 mM MgSO_2_ 7H_2_O, 0.04 mM nicotinic acid, 0.1 mM pyridoxine HCl, 0.01 mM pantothenic acid, 1 μM riboflavin, 0.3 μM thiamin HCl, 0.05 μM D-biotin, 4 mM L-glutamic acid, 1 mM L-arginine HCl, 1.3 mM L-cysteine HCl, 0.1 mM L-tryptophan, filter sterilized) [42]. For experiments that required growth on solid medium, cultures were plated on Brain Heart Infusion agar (BHI, Difco) containing the appropriate antibiotics. Cultures were grown at 37°C, 5% CO_2_, without shaking. Antibiotics were used at the following concentrations: erythromycin 10 μg/ml, tetracycline 10 μg/ml, spectinomycin 250 μg/ml, chloramphenicol 5 μg/ml, and kanamycin 250 μg/ml.

**Table 1:** Bacterial strains and primers used in this study.

Strains	Relevant characteristics	Source
*S*. *gordonii* SecCR1	*hppG*::*tet*, secretes anti-CR1 scFv, Tet^R^, Spec^R^	49
*ΔsdbA*	SecCR1, *sdbA*::*ermAM*, Tet^R^, Spec^R^, Erm^R^	20
+SdbA	*ΔsdbA*, *sdbA*-complemented on chromosome, Tet^R^, Spec^R^, Kan^R^	20
*ΔdegP*	*degP*::*ermAM*, Tet^R^, Spec^R^, Erm^R^	19
*ΔciaRH*	SecCR1, *ciaRH*::*aphA3*, Tet^R^, Spec^R^, Kan^R^	19
*ΔsdbAΔciaRH*	*ΔsdbA*, *ciaRH*::*aphA3*, Tet^R^, Spec^R^, Kan^R^, Erm^R^	19
*ΔsdbA* + CiaRH	*ΔsdbAΔciaRH*, *ciaRH*-complemented on chromosome, Tet^R^, Spec^R^, Erm^R^, Cm^R^	19
OB219	*sspAsspB*::*ermAM*, Erm^R^	53
OB219 *ΔciaRH*	*ΔsspAΔsspB*, *ciaRH*::*aphA3*, Kan^R^, Erm^R^	This study

### Biofilm formation

Biofilms were grown as described by Loo *et al*. (42), with the following modifications. *S*. *gordonii* was grown in HTVG for 12 h to an optical density (OD) of approximately 1.2 at 600 nm in 15 ml conical tubes (Sarstedt, model #62.554.205). Cells were harvested by centrifugation (3000 *x g* for 10 min) and the spent medium was discarded. The cells were then gently suspended in BM to an OD_600_ of 0.250 in the same tube. All biofilms were grown using BM. Flat bottom 24-well plates (Falcon, model #353047) were inoculated with 1 ml per well of the cell suspension, and the plates were incubated for either 3 or 24 h at 37°C, 5% CO_2_. Following incubation, the medium was removed and the wells were washed twice with 1 ml phosphate buffered saline (PBS) to remove loosely attached cells. The plates were air dried for 15 min and fixed with 10% (v/v) formaldehyde and 5% (v/v) acetic acid in PBS for 15 min. The fixing solution was removed and the wells were washed 3 times with 1 ml PBS. The biofilms were then stained with 0.5 ml of 0.1% crystal violet. After 15 min, the wells were rinsed three times and the bound stain was solubilized in 1 ml of acetone/ethanol solution (1:1). The liquid was transferred to a new microtiter plate and the optical density was measured at 600 nm in a microplate reader. The biofilm assays were carried out in triplicate with three or more separate experiments.

To test the effect of peptides on biofilm formation, biofilms were prepared as described above except that 0.5 μg of either CSP (DVRSNKIRLWWENIFFNKK; Biomatik, Cambridge, ON, Canada) or a fragment of Sth_1_ (AGFTGGIAVGLNRVNRK; Biomatik) was added to each well during the initial biofilm setup. The biofilms were then incubated for 24 h at 37°C, 5% CO_2_, and stained with crystal violet as described above.

Sensitivity to trypsin and DNase I (Sigma-Aldrich, Oakville, ON, Canada) was tested by adding the enzymes to pre-formed biofilms. Biofilms were grown for 24 h prior to the addition of 100 μg of enzyme per well. The plates were incubated at 37°C for an additional 1 h and subsequently processed and stained as described above.

To test the total growth of biofilm and planktonic cells, biofilms were removed by scraping and vigorous pipetting to remove attached cells from bottom of the well. The optical density of the combined biofilm and planktonic cells was measured at 600 nm for triplicate wells for each mutant. Crystal violet staining confirmed that the biofilms were successfully detached.

### Quantitative real-time PCR (qPCR)

Expression of *ciaR* and *degP* was measured in the biofilm inoculum and in biofilm cells. For the inoculum, RNA was isolated from cultures of the parent, *ΔsdbA*, and *sdbA-*complemented mutant grown in HTVG for 12 h to an OD_600_ of ~1.0–1.2. Total RNA was isolated using the hot acid phenol method, as described previously [54]. The RNA (1 μg) was treated with 1 unit of amplification grade DNase I (Life Technologies Inc., Waltham, MA) for 15 min at room temperature, and removal of DNA was confirmed by PCR using the 16S rRNA primers (SL525/697). cDNA synthesis was carried out using random primers and SuperScript II reverse transcriptase (Life Technologies Inc.) according to the manufacturer’s directions.

Biofilms for RNA isolation were grown in BM as described above. Cells from were suspended in BM to an OD_600_ of 0.250, and 15 ml suspensions were used to inoculate tissue culture treated petri dishes (100 mm x 20 mm, Corning, model #353003). The plates were incubated at 37°C, 5% CO_2_ for 24 h. Planktonic cells and spent medium were then removed by pipetting and the biofilms were washed once with 10 ml phosphate buffered saline (PBS). The biofilm cells were scraped from the petri dish and suspended in 1.5 ml PBS using a sterile swab. RNA isolation and cDNA synthesis was carried out as described above.

qPCR to amplify *ciaR*, *degP*, and *scaA* was carried out using the primers listed in Table 2 with iTaq Universal SYBR Green Supermix (Bio-Rad Laboratories, Mississauga, ON, Canada) according to the manufacturer’s directions. The reactions were performed using a 7900 HT Fast Real-Time PCR system (Applied Biosystems) at 95°C for 30 s, followed by 40 cycles of 95°C for 15 s and 60°C for 60 s. The cycle threshold (*C*_T_) was calculated using SDS 2.2.2 software (Applied Biosystems). The relative expression was calculated using the comparative *C*_T_ method [55] using *16S rRNA* as an internal control gene. Each reaction was performed in duplicate using cDNA from at least three biological replicates.

**Table 2:** Primers used in this study.

Primer	Gene	Direction	Description	Sequence
SL1178	*sgo*.*1071*	For	CiaRH mutant	AAAACGCTGCAAAATAATCA
SL1221	*sgo*.*1074*	Rev	CiaRH mutant	TTCAACCAATTCGCTAAATC
SL697	*16S*	For	qPCR	ATTTATTGGGCGTAAAGCGAGCGC
SL525	*16S*	Rev	qPCR	GAATTAAACCACATGCTCCACCGC
SL1214	*degP*	For	qPCR	TGGGAATAAGGTTCCTGGTG
SL1215	*degP*	Rev	qPCR	CGGCAGGAATTCTGACTACAG
SL1216	*ciaR*	For	qPCR	CATGCAGGTTTTTGATGGTG
SL1217	*ciaR*	Rev	qPCR	TCAGGAAGCATCAGATCCAG

### Genetic manipulations

A *ΔciaRH* mutant was constructed in the *ΔsspAΔsspB* mutant, OB219 [56], by creating a clean deletion of *ciaRH* and replacing the genes with a kanamycin resistance cassette (*aphA3*) [57]. Polymerase chain reaction (PCR) was carried out using Phusion high-fidelity DNA polymerase (New England Biolabs, Whitby, ON, Canada) to amplify a construct from the *ΔciaRH* mutant in the *S*. *gordonii* SecCR1 background. The construct consisted of the *aphA3* gene flanked by a 425 bp fragment of the gene upstream from *ciaR*, *sgo*.*1071*, and a 525 bp fragment of the downstream gene *sgo*.*1074*, and was amplified using the primer pair SL1178 and SL1221 (Table 2). The resulting PCR product was used to transform *S*. *gordonii* OB219 as described previously [52]. Transformants were selected on BHI containing kanamycin, and insertion of *aphA3* was confirmed by PCR.

### Carbohydrate quantification and surface charge

Total carbohydrates were measured using the phenol-sulfuric acid method [58]. Carbohydrate production was measured using cells grown for 12 h in HTVG (biofilm inoculum) and in 24 h biofilm cells. Biofilm cells were prepared from biofilms grown in petri dishes, as described above. Cells were suspended in distilled water and standardized to an OD_600_ of 2.0. The cell suspensions (200 μl) were mixed with an equal volume of 5% (vol/vol) phenol (Sigma-Aldrich), followed by the addition of 1 ml concentrated H_2_SO_4_. The reactions were incubated for 10 min at ambient temperature, cooled in a water bath for 5 min, and the optical density was measured at 490 nm. Total carbohydrates were calculated from a standard curve prepared with glucose. Surface charge was analyzed using the cationic dye Alcian Blue 8GX (Sigma-Aldrich). An Alcian Blue binding assay was carried out as described previously [59], using for cells from the biofilm inoculum and from 24 h biofilms. Briefly, the cells were washed with PBS and used to prepare aliquots standardized to an OD_600_ of 0.5. The cells were mixed with 65 μg/ml Alcian Blue and incubated at room temperature for 10 min with rotation to allow the dye to bind. The cells were removed by centrifugation at 10 000 x g, 5 min and 150 μl of supernatant was transferred to a 96-well plate. The optical density was read at 650 nm and the percentage of unbound dye was calculated as ((A_650_ Control—A_650_ Sample) / A_650_ Control) x 100%.

### Competition assay

This study was carried out in strict accordance with the Guidelines of the Canadian Council on Animal Care. The protocol was approved by the local ethics committee (University Committee on Laboratory Animals of Dalhousie University). Mice were housed in groups of 5 in conventional type II cages containing nesting materials along with water and food supply. Animal euthanasia was performed via isoflurane inhalation followed by CO_2_.

Oral colonization was tested as described previously, with modifications [60]. BALB/c mice (female, 6 weeks old, Charles River Laboratory, St. Constant, Qc, Canada) were fed aqueous kanamycin (500 μg/ml) for 2 days prior to colonization to reduce background levels of natural microflora. The *S*. *gordonii* parent and *ΔsdbA* mutant were grown for 18 h and then dechained by vigorously passing the cells through a 26 G needle. Microscopy was used to confirm dechaining. Cultures were standardized by their optical density, and the parent and the mutant were then mixed in a 1:1 ratio (50 μl total volume). The inoculum was also plated for enumeration, and contained 5.2 x 10^9^ CFU/ml of the parent and 1.8 x 10^9^ CFU/ml of the *ΔsdbA* mutant.

The mice were sedated with ketamine-xylazine and inoculated with 10 μl intranasally and 40 μl orally. After 24 h (n = 4) and 7 days (n = 6), the mice were euthanized. Sterile swabs were used to recover bacteria from the oral cavity by swabbing the tooth surfaces, tongue, buccal mucosa, and throat to the trachea. The swabs were then vortexed with 1 ml PBS and serially diluted. Aliquots were plated on selective agar that allowed for differentiation of the parent and mutant. All media included spectinomycin and tetracycline to select for the *S*. *gordonii* parent and *ΔsdbA* mutant, while preventing growth of other bacteria. To differentiate the parent and the *ΔsdbA* mutant, bacteria were plated on media with and without erythromycin. The *ΔsdbA* mutant was quantified as the erythromycin resistant colonies, while the parent was quantified by subtracting the erythromycin resistant colonies from the total CFU. The recovered CFU were standardized to the initial inoculum density, and data were analyzed by Student’s *t*-test.

### Statistical analysis

Results were analyzed by one-way ANOVA or student’s *t* test using GraphPad Prism version 6 (GraphPad Software Inc., La Jolla, California).

## Supporting Information

S1 Fig Enhanced biofilm formation by *S*. *gordonii ΔsdbA* and *ΔsspAB* mutants involves different mechanisms. (a) Quantitative PCR analysis of *scaA* expression in 24 h biofilm cells. RNA was prepared from the parent, the *ΔsdbA* mutant, and the *sdbA*-complemented mutant (+SdbA). (b) Biofilm formation by the parent, the *ΔsdbA* mutant, and the *ΔsspAB* mutant (OB219) with and without a functional CiaRH two-component signaling system. Biofilms were grown for 24 h and stained with crystal violet.
(TIFF)
