# Supplementary figures and images for: Mutation of the Streptococcus gordonii Thiol-Disulfide Oxidoreductase SdbA Leads to Enhanced Biofilm Formation Mediated by the CiaRH Two-Component Signaling System
Source: PLoS One. 2016 Nov 15;11(11):e0166656. doi: 10.1371/journal.pone.0166656 (PMC5112981; doi:10.1371/journal.pone.0166656)

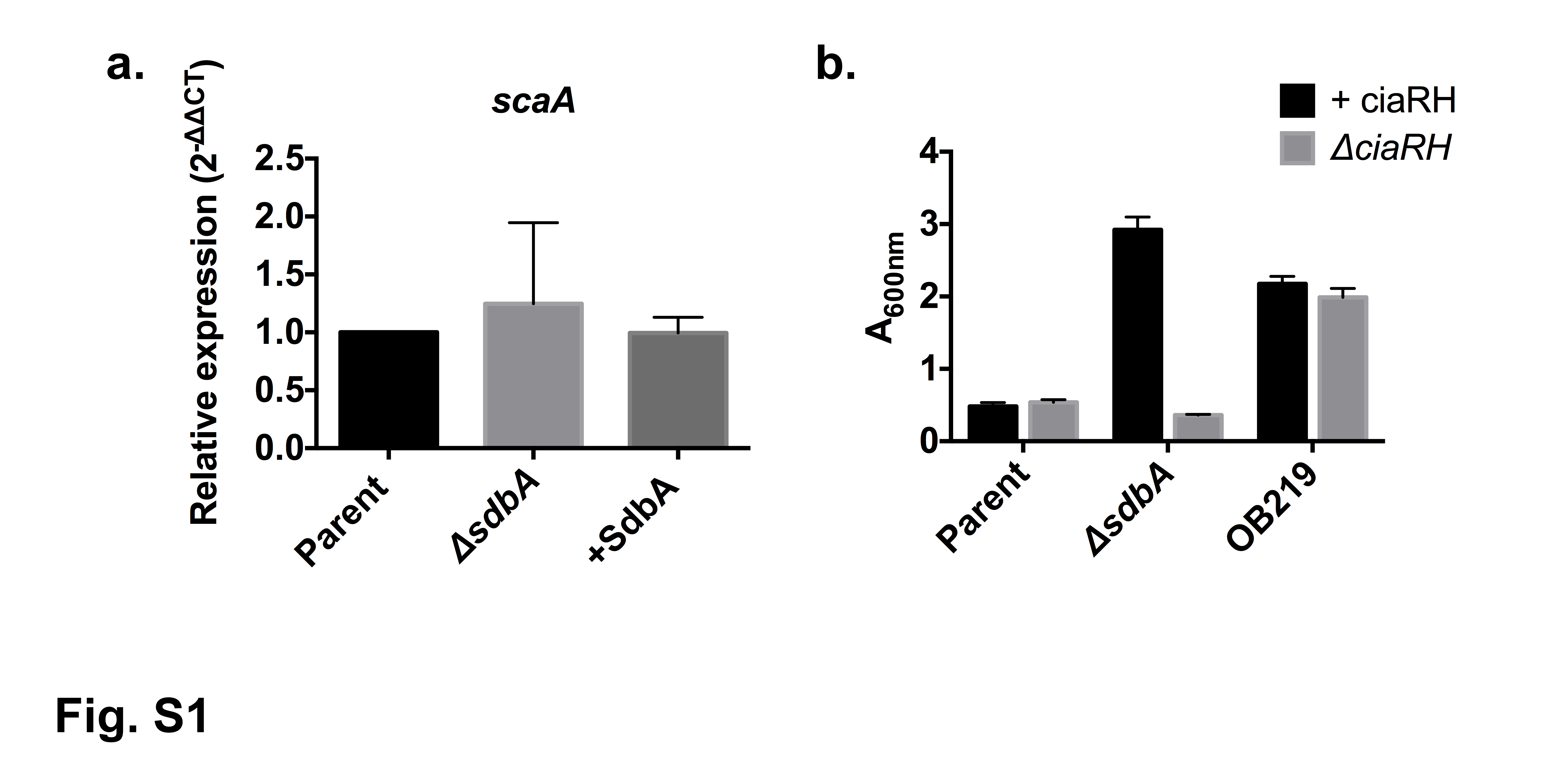

Supplement: S1 Fig — (a) Quantitative PCR analysis of scaA expression in 24 h biofilm cells. RNA was prepared from the parent, the ΔsdbA mutant, and the sdbA-complemented mutant (+SdbA). (b) Biofilm formation by the parent, the ΔsdbA mutant, and the ΔsspAB mutant (OB219) with and without a functional CiaRH two-component signaling system. Biofilms were grown for 24 h and stained with crystal violet. (TIFF) [file pone.0166656.s001.tiff]
